# How safe is the use of chlorpyrifos: Revelations through its effect on layer birds

**DOI:** 10.14202/vetworld.2016.753-758

**Published:** 2016-07-22

**Authors:** P. P. Singh, Ashok Kumar, R. S. Chauhan, P. K. Pankaj

**Affiliations:** 1 Section of Animal Science, KVK, Rajmata Vijayaraje Scindia Krishi Vishwavidyalaya, Morena, Madhya Pradesh, India; 2 Department of Livestock Production and Management, College of Veterinary & Animal Sciences, Govind Ballabh Pant University of Agriculture and Technology, Pantnagar, Uttarakhand, India; 3 Section of Transfer of Technology, Livestock Production Management, ICAR-Central Research Institute for Dryland Agriculture, Hyderabad, Telangana, India

**Keywords:** blood biochemistry, chlorpyrifos, layers, immunity, organic pollutant

## Abstract

**Aim:**

The present study was aimed to investigate the immunological competence of chlorpyrifos (CPF) insecticide after oral administration in layer chickens.

**Materials and Methods:**

A total of 20 White Leghorn birds were given CPF in drinking water at 0.3 ppm/bird/day (no observable effect level dose) for a period of 3-month. Immune competence status of layer birds and chicks hatched from CPF-treated birds were estimated at 15 days interval in layer birds and monthly interval in chicks using immunological and biochemical parameters.

**Results:**

There was a significant decrease in values of total leukocytes count, absolute lymphocyte count, absolute heterophil count, total serum protein, serum albumin, serum globulin, and serum gamma globulin in the birds treated with CPF as compared to control. Similarly, immune competence tests such as lymphocyte stimulation test, oxidative burst assay, and enzyme-linked immunosorbent assay tests indicated lower immunity in birds treated with CPF as compared to control. Subsequently, chicks produced from CPF-treated birds were also examined for immune competence, but no significant difference was observed between chicks of both the groups.

**Conclusion:**

The exposure to CPF produced hemo-biochemical and other changes that could be correlated with changes in the immunological profile of layer chickens suggesting total stoppage of using CPF in poultry sheds.

## Introduction

Chlorpyrifos (CPF) (O,O-diethyl O-3,5,6-trichloro-2-pyridyl phosphorothioate), particularly affects the cholinesterase system [1], is a currently most widely used broad-spectrum chlorinated organophosphate insecticide [2] in agriculture worldwide. Poultry farming in India is and internal part of the agricultural industry [3,4]. CPF has been used for the control of termites in chicken houses [5]. Organophosphate insecticides are increasingly used as substitutes for organochlorine and carbamate insecticides because of their high efficacy and lower persistence in the environment [2,5,6]. Thus, the present topic is important to support the discontinuance of use of organophosphates in the vicinity of poultry houses.

### Significance of the study

Toxicological studies [2] of CPF in chickens focused on the sub-acute effects on plasma or serum enzymes and other biochemical parameters, foodborne toxicity [7], developmental effects [8], and pathology of long-term exposure [9]. Hematological, biochemical, and pathological effect of chronic exposure to CPF was also evaluated in indigenous chickens [10-13]. The continuous use of pesticides even in normal recommended doses may cause deleterious effect on the physiological functions [14-16] that may range from lower immunity of flock [17,18] to decreased production performances. The lower immune competence in animals and birds due to environmental pollutant may lead to increase susceptibility [19,20], occurrence of re-infection, epidemics of disease [5,21,22], and vaccine failures [6] causing serious economic losses and thereby hampering the purpose of raising animals and birds.

### Aim of the study

The present study was undertaken with the aim to study the effects of CPF on immunity in layers and subsequently, its effect on immune status and other exhibits in chicks produced from these organophosphate treated birds.

## Materials and Methods

### Ethical approval

The experiments were performed after obtaining permission from the Animal Ethics Committee. The regulations addressing animal use were followed and proper care and attention were given to the birds used in these experiments. Moribund birds and the birds at the end of the experiments were humanely sacrificed by cervical dislocation.

### Experimental design

About 20 White Leghorn layer birds of around 1 year age were selected for the present study at Poultry Research Centre, Govind Ballabh Pant University of Agriculture and Technology, Pantnagar. All the birds were maintained under standard and uniform conditions of feeding, management, and disease control under deep litter system of housing. The selected birds were vaccinated with Ranikhet vaccine F_1_ strain through intraocular route for both primary and booster dose. The chicks hatched out of CPF-treated layer birds were also assessed for immunocompetence tests.

The CPF (20% E.C.), named Perfban from Perfect Cropscience Pvt. Ltd., Ahmedabad, India, was procured from local market and mixed in drinking water. A solution was prepared for CPF by taking CPF 10 ml original solution and mixed in 90 ml of water, and from this solution, 1.5 ml was daily mixed in 100 ml of drinking water of birds (T_1_) to give them desired concentration of 0.3 ppm per bird. Control (C) birds were given normal water. All the birds under study were provided 12 h of light period uniformly. The experiment was divided into two parts:

### Effect of CPF in layers

In the first part of the experiment, 20 birds were divided at random into two groups, *viz*., control (C) and CPF-treated group (T_1_), each having 10 birds comprising of 8 hens and 2 cocks. Immunological and biochemical parameters in these layers were studied after collection of blood from these birds from wing vein at 15 days interval up to 90 days. The first moiety of blood was taken in heparinized vials (10-12 IU/ml) to study the biochemical parameters (serum total protein, albumin, globulin, and gamma globulin). The second moiety of blood was collected in sterilized vials for the collection of serum and further carrying out immunological studies and antibody titer through enzyme-linked immunosorbent assay (ELISA) using Ranikhet disease virus antigen [23]. The following immunological studies were carried out:

- Lymphocyte blastogenesis assay using Con-A and lipopolysaccharide as mitogen [23],
- Total leukocytes count (TLC), absolute lymphocyte count (ALC), and absolute heterophil count (AHC) [24].
- Oxidative burst assay for macrophage activity [25].

### Effects of CPF in chicks hatched from CPF-treated birds

The second part of the experiment included the study of immune competence status of chicks obtained from CPF-treated birds. These chicks were divided into three categories, i.e., the chicks out of birds treated with CPF for 1, 2, and 3 months.

All the chicks under each category were maintained for 1 month, and at the end, blood samples from all the chicks were collected directly from the heart into two parts. The first moiety was taken in heparinized vials to assess the hematological parameters and another moiety was taken in sterilized vials for serum collection and further assessment of immunological parameters.

The hematological/immunological and biochemical parameters studied in layers to assess the effect of CPF and were also studied in these chicks. None of the chicks were treated individually with CPF.

### Statistical analysis

All data were expressed as mean ± standard error. The statistical significance of the mean differences between control and treated groups was analyzed by Student’s *t*-test [26]. Statistical calculations were performed with the SPSS 13 computer program (SPSS Inc., Chicago, Illinois, USA). The value of p<0.05 was taken as the cut-off value to consider differences statistically significant.

## Results and Discussion

The CPF pesticides were found to have a depressive effect on total leukocytes and absolute lymphocyte counts after feeding for 90 days. These cells were found to be decreased up to 9.55% as compared to control (Table-1). Leukopenia may be due to cytotoxic effects of CPF; since the lymphocytes are the main cells to play the key role in defense mechanism, a reduction in the number of absolute lymphocytes as observed in the present study is an indication of immunosuppression. Earlier studies with lindane, quinalphos, carbaryl, fenvelerate, butachlor, and isoproturon revealed leukopenia and lymphopenia [27-29].

**Table-1:** Effect of CPF (mean±SE) on various leukocytic counts (10^3^/µl) in layers.

Days	TLC		ALC		AHC	
		
	C	T_1_	C	T_1_	C	T_1_
0	24.53±0.55	24.53±0.55	15.43±0.45	15.20±0.44	7.84±0.17	8.08±0.14
15	26.10±0.27	24.14±0.23*	16.18±0.12	15.69±0.19	7.83±0.21	7.39±0.66
30	27.30±0.22	25.22±0.21*	18.55±0.89	16.61±0.61*	7.53±0.52	7.30±0.50*
45	28.99±0.19	26.11±0.11*	20.20±0.22	17.78±0.79*	7.53±0.15	6.96±0.94*
60	30.29±0.20	28.16±0.17*	22.49±0.32	18.79±1.17*	6.66±0.46	6.75±0.20
75	31.04±0.13	28.78±0.30*	23.59±0.13	20.81±0.14*	5.83±0.11	6.51±0.48
90	31.93±0.31	28.88±0.32*	25.01±0.28	20.60±0.15*	6.82±1.14	7.02±0.47

* Significant difference from control within column (p≤0.05). TLC=Total leukocyte counts, ALC=Absolute leukocyte counts, AHC=Absolute heterophil count, CPF=Chlorpyrifos, SE=Standard error

Birds immunized with Ranikhet disease vaccine and exposed to CPF showed marked decrease in delta optical density after stimulation with mitogen Con-A, which indicates lowered cellular immune response. Decrease in the lymphocyte proliferation by mitogen Con-A (17.88% Table-2) is an indication of suppression of T-cell blastogenesis, which is essential for mounting of both the cell-mediated and humoral immune response. Earlier, workers have also reported the [30] reduced lymphocyte blastogenesis response in minks and ferrets exposed to hexachlorobenzene. Depression in cell-mediated immune response in pesticide offered birds, as observed in this study has also been reported in broilers as measured by lymphocytes migration inhibition test [31] and lymphocyte stimulation test (LST) using 3-(4,5-dimethylthiazol-2-yl)-2,5-diphenyltetrazolium bromide dye method [27,29,32].

**Table-2:** Effect of CPF (mean±SE) on cell-mediated and humoral immune response in layers.

Days	Cell-mediated immune response				Humoral immune response			
	
	T-lymphocyte blastogenesis (delta OD)		OBA (NO-_2_ production µM/ml)		Humoral immunity (ELISA values)		B-lymphocyte blastogenesis (delta OD±SE)	
			
	C	T_1_	C	T_1_	C	T_1_	C	T_1_
0	0.201±0.025	0.201±0.025	190.40±22.78	190.40±22.78	1.52±0.26	1.52±0.26	0.160±0.090	0.160±0.090
15	0.189±0.015	0.194±0.024	215.20±30.70	126.03±23.13	2.55±0.06	2.52±0.15	0.197±0.076	0.181±0.046
30	0.207±0.045	0.198±0.046	346.28±33.12	257.04±31.70	2.45±0.04	2.48±0.05	0.252±0.016	0.199±0.035
45	0.255±0.025	0.220+0.025	181.96±26.72	107.33±12.07	2.40±0.09	2.29±0.06	0.317±0.057	0.246±0.075*
60	0.285±0.055	0.229±0.045*	311.31±36.98	223.67±32.49*	2.30±0.07	2.07±0.02*	0.330±0.046	0.257±0.052*
75	0.291±0.043	0.238±0.036*	132.36±22.53	25.02±5.44*	2.29±0.08	2.06±0.04*	0.337±0.089	0.252±0.044
90	0.302±0.059	0.248±0.082*	316.04±34.61	223.64±33.03*	2.25±0.05	2.06±0.03*	0.345±0.023	0.262±0.024

* Significant difference from control within column (p≤0.05). OBA=Oxidative burst assay, OD=Optical density, ELISA=Enzyme-Linked immunosorbent assay, CPF=Chlorpyrifos, SE=Standard error

In the present investigation, the production of nitric oxide in avian macrophage culture was found to be reduced as a result of CPF treatment. The reduction of nitrous oxide (NO) varied in a dose-dependent manner. When macrophages are activated with bacterial lipopolysaccharides, the expression of high levels of nitric oxide synthetase occurs that oxidized L-arginine to L-citrulline and NO gas [33]. NO is a gaseous, free radical molecule, which is catalytically generated by the cellular NO synthetase from L-arginine to L-citrulline. Besides, being an essential neurotransmitter, NO or its derivatives have inhibitory effects on a variety of infections [34]. NO has potent antimicrobial activity and even it can combine with superoxide anion to yield more potent antimicrobial activity against, bacteria, fungus, and other pathogens [35]. The reduction in NO production as revealed in the present study, i.e., 29.33% (Table-2) in CPF-treated birds lead to a state of decreased phagocytosis and immunosuppression.

Significant (p<0.05) depression in Ranikhet disease vaccine-induced humoral immune response as measured by ELISA has been observed in the present study in birds exposed to CPF as compared to control. The decrease in ELISA values (8.44% Table-2) was observed in birds treated with CPF as compared to control. The immunosuppressive action of pesticides is due to their detrimental action on lymphoid organs. The decreased number of lymphocytes along with decreased functional capacity has been reported in patients following pesticide intoxication [36]. The decreased activity of T-lymphocytes might affect T-cell dependent humoral immune response also.

The reduction in antibody titer was further confirmed by decreased serum gamma globulins and decreases the activity of B-lymphocyte blastogenesis by 24.05% (Table-2) in CPF-treated birds. The gamma globulins are directly related to the antibody titer measured by ELISA, which was found to be significantly decreased in the CPF-treated group. The 42.85% decrease in serum gamma globulin (Table-2) is an indication of lowered immunity while ELISA detected the reduction in specific antibody titer to Ranikhet disease vaccine. A decrease in serum globulin and immunoglobulin levels has been reported in animals [37] and rats due to lindane [38] in birds due to carbaryl, malathion [31], cypermethrin [32], gamma benzene hexachloride and quinalphos [27], and butachlor and isoproturon [39]. Alteration in immunoglobulins levels was reported in offsprings of mice as a result of prenatal exposure of carbofuran or diazinon [39]. The decrease in serum gamma globulin contents might be due to the effect of pesticides on peripheral blood lymphocytes, which were found to be decreased in the present study. Since, the gamma globulins are synthesized by the lymphocytes and pesticides being lymphocytotoxic, the quantitative reduction was evident because of their effect on peripheral blood lymphocytes [5]. Reduction in serum gamma globulin concentration might also be due to the immunosuppressive effect of insecticides on antibody production or due to functional impairment of B-lymphocytes [40-42].

The total serum proteins, serum albumin, and serum globulin values were significantly less in CPF-treated birds as compared to control birds (Table-3). Similarly, decreased serum proteins were also reported in Malathion fed birds [5,31] and goats fed carbaryl [43]. A decrease in serum proteins was reflected due to decrease in albumin and gamma and beta globulin values in rabbits administered dichlorodiphenyltrichloroethane [44] and in buffalo calves sprayed with deltamethrin [45].

**Table-3:** Effect of CPF (mean±SE) on serum proteins in layers.

Days	Serum albumin (mg/dl)		Serum globulin (mg/dl)		Gamma globulin (g/100 ml)		Serum total proteins (g/100 ml)	
			
	C	T_1_	C	T_1_	C	T_1_	C	T_1_
0	2.50±0.31	2.50±0.31	1.87±0.32	1.87±0.32	1.30±0.32	1.30±0.32	4.37±0.18	4.37±0.18
15	2.27±0.34	2.00±0.17	2.23±0.19	2.00±0.17	1.56±0.17	1.40±0.27	4.50±0.18	3.90±0.18*
30	2.35±0.17	2.10±0.29	2.65±0.20	2.00±0.35*	1.85±0.37	1.30±0.17*	5.00±0.20	4.00±0.46*
45	2.40±0.32	2.15±0.17*	2.90±0.27	1.95±0.27*	2.03±0.40	1.32±0.27*	5.30±0.13	4.20±0.37*
60	2.70±0.32	2.30±0.21*	2.30±0.39	2.00±0.31*	1.61±0.36	1.36±0.19	6.00±0.32	4.19±0.32*
75	2.90±0.09	2.40±0.19*	3.60±0.22	2.10±0.39*	2.52±0.19	1.44±0.21	6.50±0.36	5.00±0.40*
90	3.00±0.19	2.35±0.28*	4.00±0.17	2.35±0.60*	2.80±0.22	1.60±0.32*	7.00±0.50	5.32±0.41*

* Significant difference from control within column (p≤0.05). CPF=Chlorpyrifos, SE=Standard error

In the present study, the results on the effect of CPF on chicks hatched from CPF-treated birds revealed the non-significant difference between groups for all the hematological, biochemical, and immunological parameters studied (Tables-4 and 5). The values for all the hematological parameters TLC, ALC, and AHC were approximately similar in control and treated groups of chicks. Similarly, the values for biochemical parameters serum total proteins, albumin, globulin, and gamma globulins were also approximately same for control and CPF-treated groups. The values for immunological status (LST and ELISA) were also approximately similar in control and CPF-treated group of chicks. Similar results have been observed in interactive research with CPF and other elements [34,35,46].

**Table-4:** Effect of CPF (mean±SE) on Cell-mediated and humoral immune response in chicks.

Months	Cell-mediated immune response		Humoral immune response			
	
	Tlymphocyte blastogenesis (delta OD)		Blymphocyte blastogenesis (delta OD)		Humoral immunity (ELISA values)	
		
	C	T_1_	C	T_1_	C	T_1_
1^st^	0.20±0.27	0.27±0.19	0.150±0.32	0.090±0.31	0.74±0.14	0.64±0.27
2^nd^	0.29±0.32	0.26±0.17	0.350±0.17	0.340±0.28	0.56±0.41	0.65±0.60
3^rd^	0.27±0.18	0.25±0.20	0.094±0.26	0.090±0.38	0.63±0.10	0.70±0.80

ELISA=Enzyme-Linked immunosorbent assay, CPF=Chlorpyrifos, SE=Standard error

**Table-5:** Effect of CPF (mean±SE) on various leukocytic counts (10^3^/µl) in chicks.

Months	TLC		ALC		AHC	
		
	C	T_1_	C	T_1_	C	T_1_
1^st^	22.39±0.34	21.33±0.68	13.80±0.27	13.01±0.50	7.01±0.62	6.60±0.15
2^nd^	21.32±0.52	20.84±0.34	13.37±0.17	12.78±0.16	6.77±0.53	6.73±0.12
3^rd^	21.40±0.61	20.91±0.45	13.04±0.25	12.75±0.30	6.63±0.31	6.47±0.71

TLC=Total leukocyte counts, ALC=Absolute leukocyte counts, AHC=Absolute heterophil count, CPF=Chlorpyrifos, SE=Standard error

## Conclusion

It can be concluded that there were leukopenia and lymphopenia in CPF-treated birds with decreased number of heterophils. Similarly, there was a decrease in cellular and humoral immune response in CPF-treated birds. Total serum protein, serum albumin, serum globulin, and serum gamma globulin were significantly decreased in CPF-treated birds. Antibody titer against Ranikhet disease vaccine was decreased in CPF-treated birds as compared to control. The findings in the present study are suggestive of very damaging effects of CPF even at no observable effect level dose in layer birds. Elaborate work needs to be conducted on a large scale before recommending the further use of this organophosphate in the poultry sheds.

## Authors’ Contributions

PPS, AK, and RSC designed the work. PPS conducted the study. PPS and PKP helped for statistical analysis. PPS and PKP prepared the manuscript. PKP revised the manuscript for communication to the journal. All authors read and approved the final manuscript.
